# Improving the Quality and Completeness of Discharge Summaries at a Tertiary Care Hospital in Pakistan: A Quality Improvement Project

**DOI:** 10.7759/cureus.56134

**Published:** 2024-03-14

**Authors:** Faizan Fazal, Maham L Adil, Talha Ijaz, Shahrukh Ahmad Khan, Ahmed Imran Butt, Areesha Abid, Muhammad N Bashir, Saima Ambreen, Taha Z Chaudhry, Bilal H Malik

**Affiliations:** 1 Department of Medicine, Holy Family Hospital, Rawalpindi, PAK; 2 Department of Internal Medicine, Rawalpindi Medical University, Rawalpindi, PAK; 3 Department of Internal Medicine, California Institute of Behavioral Neurosciences & Psychology, Fairfield, USA

**Keywords:** quality improvement projects, hospital discharge, level of completeness, health communication, clincal audit

## Abstract

Introduction

Discharge summaries (DS) allow continued patient care after being discharged from the hospital. Only a few quality improvement projects (QIPs) focused on assessing and improving the quality and completeness of DS at tertiary care hospitals have been undertaken in Pakistan. This QIP aimed to evaluate and enhance the quality and completeness of DS at a tertiary care hospital in Pakistan to facilitate seamless healthcare transitions.

Methods

A QIP was conducted in the medical unit of a tertiary care hospital in Rawalpindi, Pakistan. The DS were assessed using the e-discharge summary self-assessment checklist devised by the Royal College of Physicians (RCP). This QIP was done by the plan, do, study, act (PDSA) cycle. The PDSA cycle comprised two audit cycles and an intervention in between them. The first audit cycle (AC) was conducted on 150 DS. Its duration was from March 2023 to June 2023. An educational workshop was conducted before the re-audit cycle (RAC) to address deficiencies and reinforce the implementation of the guidelines provided by the RCP. The RAC was conducted from June 2023 to August 2023. 100 DS were studied and analyzed to assess for improvement in the completeness of DS. Frequencies and percentages were calculated in each audit cycle. The Chi-squared test was applied to compare the statistical difference between the results of both audit cycles.

Results

A total of 150 DS were analyzed in the first AC and 100 DS in the RAC. The results of the first AC show that the details of any allergies were recorded only in 3% of the DS; this percentage significantly improved to 51% after the RAC (p-value <0.05). Relevant past medical history was included in 52% and 88% of the DS during the first AC and RAC, respectively (p-value <0.05). Secondary diagnoses were written in 54% and 71% of the DS during the first AC and RAC, respectively (p-value <0.05). Details of relevant investigations were included in 60% and 88% of the DS during the first AC and RAC, respectively (p-value <0.05). The post-discharge management plan was written in 90% and 98% of the DS during the first AC and RAC, respectively (p-value <0.05). The follow-up plan was written clearly in 65% and 93% of the DS during the first AC and RAC, respectively (p-value <0.05).

Conclusion

The DS was found to be incomplete after analyzing the results of the first AC. The details related to allergies, medications, operations, and procedures were found to be missing in the majority of the cases. No mention of the patient's concerns or expectations was made in the DS. The results of the RAC showed improvement in the level of completeness of DS. The majority of the weak points observed after the first AC seemed to have improved after the RAC, which shows that intervention proved to be quite effective in improving the completeness and quality of DS. The RAC showed significant improvement in the completeness of the details relating to investigations, allergies, past medical history, secondary diagnoses, and the post-discharge follow-up plan. QIP must be routinely carried out to assess and improve the completeness and quality of DS at hospitals.

## Introduction

Efficient and complete communication in healthcare is essential for optimum care provision to patients. One such form of communication is hospital discharge summaries, which are described as a synopsis of all the events that occurred during a patient's hospital stay, outlining all relevant information [1]. Timely and high-quality hospital discharge summaries are crucial for ensuring patient safety and facilitating efficient healthcare services following discharge. Various systematic reviews have been conducted to find the most integral components of a discharge summary and revealed that the most essential components are the reason for admission, treatment received during hospital stay, outstanding results on discharge, the primary diagnosis, and subsequent management [2,3]. The discharge summary is the primary method of information transfer between various healthcare providers. Most discharge summaries are created with primary care providers in mind [4]. As essential as a well-written summary is, the threats a bad-quality discharge summary poses are immense. One study suggests that the most common cause of medical errors in a clinical setting is bad communication [5]. Missing important information or getting incorrect information leads to unnecessary delays in treatment [6].

The most ideal way to standardize the process of discharge summaries is to follow a standard template, which will minimize the chances of missing out on important information from the summary, reduce human error, and ensure consistency of documentation across various healthcare settings [7]. In Pakistan, electronic discharge summaries are slowly being adopted. There is a lack of proper documentation across all aspects of healthcare, specifically where discharge summaries are concerned. This, coupled with the lack of information among patients about their own disease and treatment, creates an unfavorable situation for follow-up visits and the continuation of patient care after discharge from the hospital. In the United Kingdom, the Standards for the Clinical Structure and Content of Patient Records of the Academy of Medical Royal Colleges (AoMRC) have been adopted by the Professional Record Standards Body (PRSB). They have developed standards for the discharge summary, which should include the following details: the patient's personal information (name, identification number, date of birth, address, and any special needs); the general practitioner's information (name and address); admission information (date, source, method, and reason); a summary of the clinical encounter; procedures and surgeries performed; investigations and their results; changes in medication; and details of discharge medications, allergies, risks, and warnings, diagnoses given at the time of discharge, discharge specifics (date, consultant, department, location, and destination), advice, recommendations, and future plans (including follow-up details), legal information and capacity, concerns, wishes, and expectations of the patient and their caregiver, as well as the name, grade/designation, and signature of the attending doctor responsible for completing the discharge summary [6]. 

Clinical audits are an effective way of ensuring adherence to standards in a healthcare setting and improving the quality of services being provided [8]. Holy Family Hospital is a public hospital located in the center of Rawalpindi, Pakistan. In Holy Family Hospital, the discharge summaries are made manually. No set template is followed. We conducted a clinical audit to assess the quality of the discharge summaries made in Medical Unit 1, Holy Family Hospital. This consisted of two audit cycles with interventions between the cycles to address the gap and encourage improvements. To the best of our knowledge, no similar audit has been conducted in this clinical setting previously.

## Materials and methods

Study setting and ethical approval

This quality improvement project (QIP) was conducted in the medical unit of Holy Family Hospital (HFH), Pakistan. Ethical permission was taken from the institutional review board of Rawalpindi Medical University, Pakistan. The ethical approval reference number is 28-MED-46-23.

Study objective

This QIP was conducted to assess the completeness and quality of discharge summaries (DS). The objective was to assess the completeness of different aspects of DS. Re-audit was conducted to ensure that deficiencies observed in the first audit cycle (AC) were addressed.

Eligibility criteria

The inclusion criteria were discharges produced during the 12 weeks before the first AC in the inpatient department of the medical unit of HFH. Exclusion criteria were discharges produced in the emergency department.

The plan, do, study, act (PDSA) cycle

**Figure 1:**
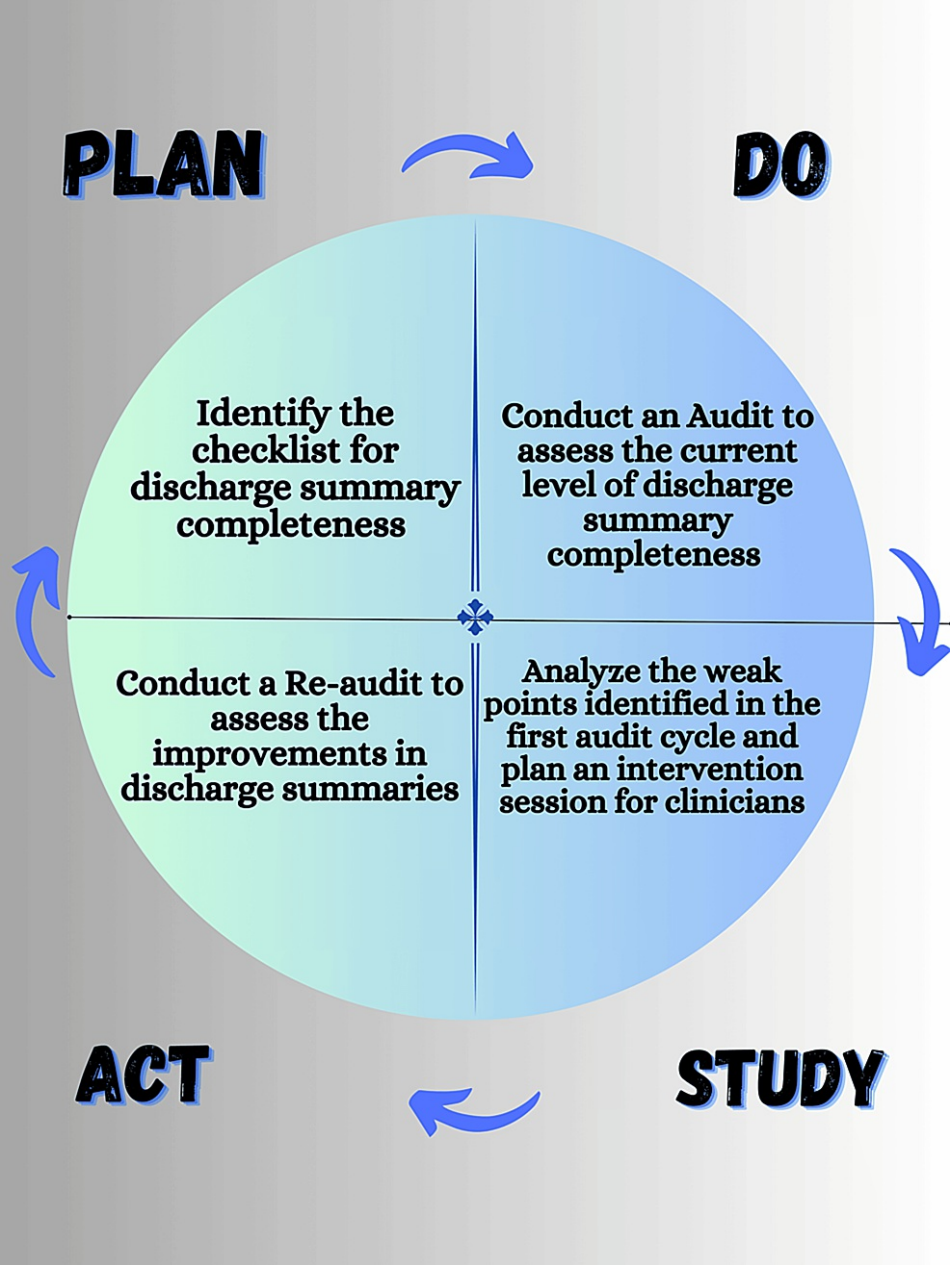
The plan, do, study, act (PDSA) cycle

This QIP was done by the plan, do, study, act (PDSA) cycle. The PDSA cycle comprised two audit cycles and an intervention in between them. The first AC was conducted on 150 DS. Its duration was from March 2023 to June 2023. After completing the first AC, an analysis was done to assess the completeness of DS. Several points were observed after analyzing the results of the first AC, which required special attention. An intervention session was arranged for the healthcare staff based on the analysis of the first AC. The healthcare staff was briefed on the aspects of DS that were incomplete or absent. The findings of the first AC were presented in the morning meeting with the entire department's doctors present in the meeting. A detailed discussion was held in which emphasis was laid upon the main areas where the department's DS was found to be lacking. The e-discharge summary standard was discussed, and the different components of a complete discharge were emphasized. The re-audit cycle (RAC) was conducted after the intervention session from June 2023 to the start of August 2023. 100 DS were studied and analyzed in the RAC to assess for improvement in the completeness of DS. The PDSA cycle is shown in Figure 1. 

Data analysis

The data was then evaluated using Microsoft Excel (version 2019; Microsoft, Redmond, Washington) and SPSS (IBM Inc., Armonk, New York). The study utilized descriptive statistics, including percentages and frequencies, to describe the extent and comprehensiveness of the discharge summary information. The Chi-squared test was also applied to the various components of the DS to assess the statistical difference between the results of the first AC and RAC. The findings were then represented through figures and tables.

Study questionnaire

**Figure 2:**
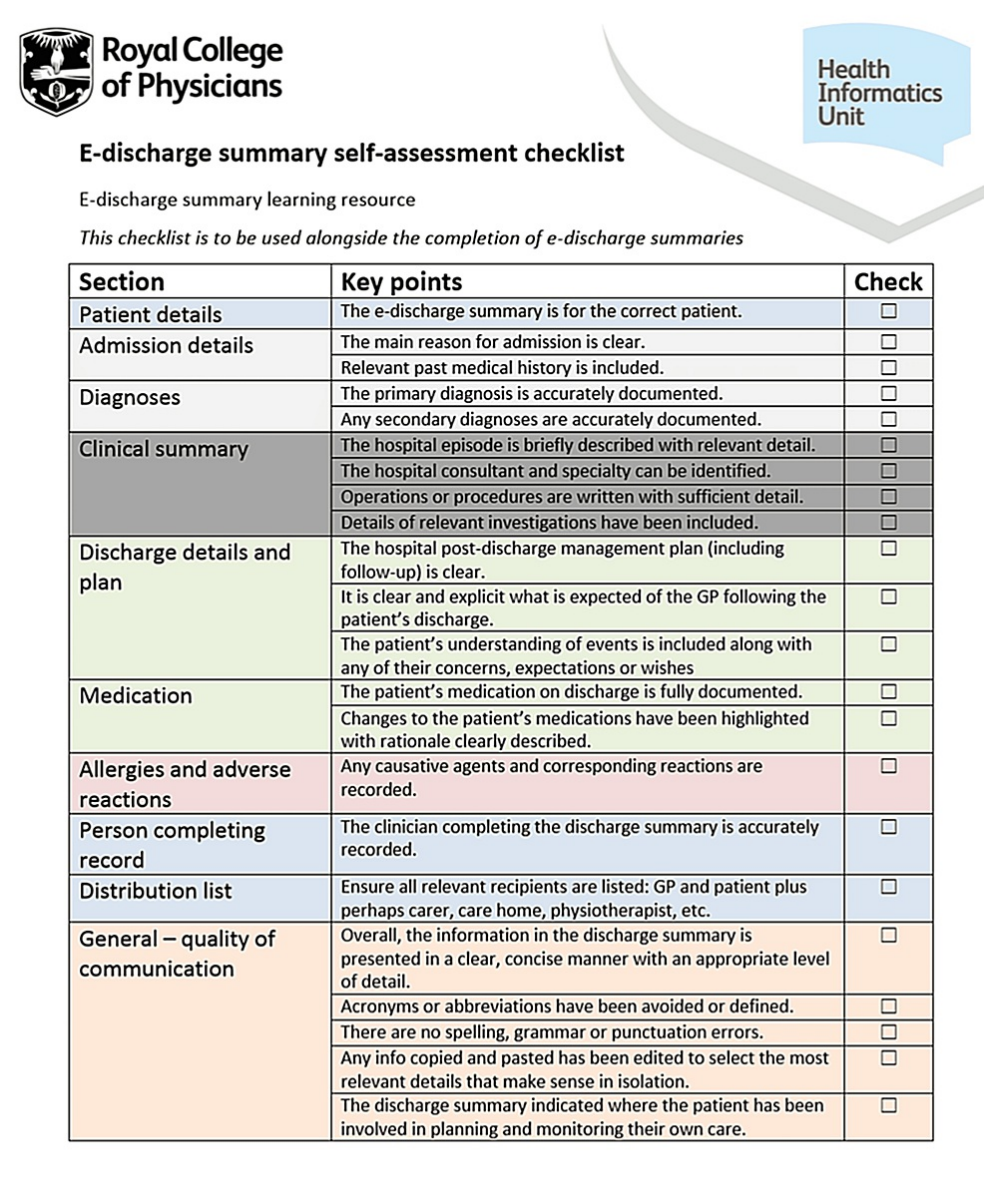
The e-discharge summary self-assessment checklist devised by the Royal College of Physicians

The e-discharge summary self-assessment checklist devised by the Royal College of Physicians was used to assess the completeness of DS during both audit cycles. This checklist consists of 11 sub-sections on various aspects of the DS. It covers all the important and relevant aspects of the DS that the healthcare staff needs to fill while writing the discharges of the patients. An audit template was created using Google Sheets (Google, Mountain View, California) containing 25 questions divided into 11 sections using the e-discharge summary standard as a reference. The 11 sections that the form was divided into were: filling in doctor's details, patient's details, admission details, diagnoses, clinical summary, discharge details and plan, medication, allergies and adverse reactions, person completing record, distribution list, and general/quality of communication. The responses were recorded as one of the following: 'Yes', 'No', or 'Maybe', except for 'filling doctor's details'. During this QIP, the researchers recorded the completeness of DS by comparing them with the Royal College of Physicians checklist. The e-discharge summary self-assessment checklist is attached in Figure 2.

## Results

Quality of information in discharge summaries (DS) observed in the first audit cycle (AC)

**Figure 3:**
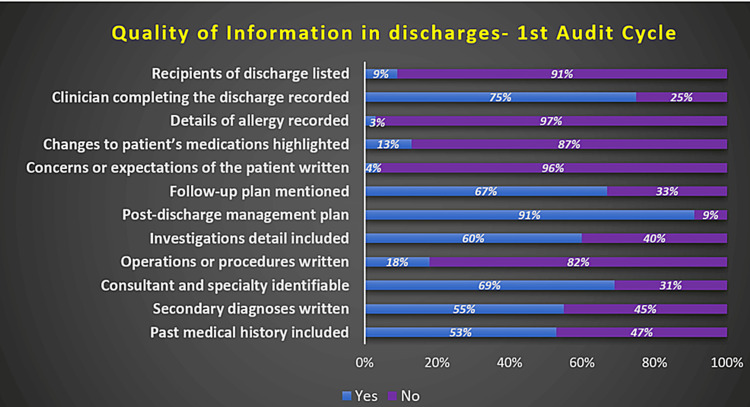
Quality of information in discharge summaries observed in the first audit cycle

During the first AC of this Quality improvement project (QIP), it was observed that the recipients were only listed in 9% of the DS. Details of any allergies were recorded only in 3% of the DS. Changes to the medications of the patients were only mentioned in 13% of the DS. Concerns and expectations of the patients were mentioned in only 4% of the DS. The post-discharge management plan was written in 91% of the DS. Patients' past medical history was only written in 53% of the DS. The completeness of the rest of the components observed in the first audit cycle (AC) is depicted in Figure 3. 

Quality of information in discharge summaries observed in the re-audit cycle (RAC)

**Figure 4:**
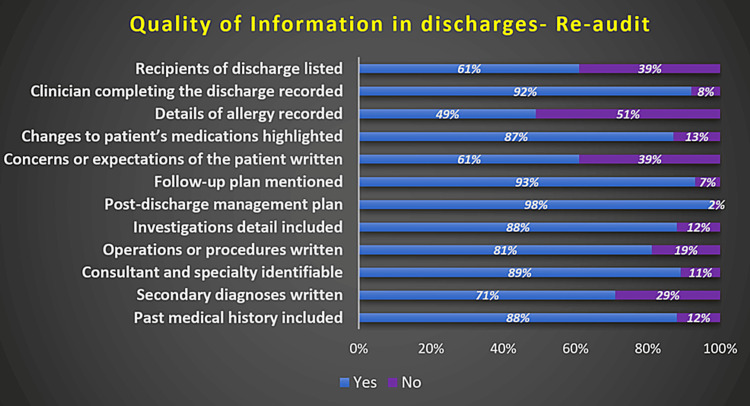
Quality of information in discharge summaries observed in the re-audit

The clinician completing the record was recorded in 92% of the DS. Changes made to the patient's medications were highlighted in 87% of the DS. The follow-up plan was mentioned in 93% of the DS. Details of any procedure or operations were mentioned in 81% of the DS. Figure 4 shows the completeness of various aspects of DS observed in the re-audit cycle (RAC).

Quality of communication in DS observed in the first AC

**Figure 5:**
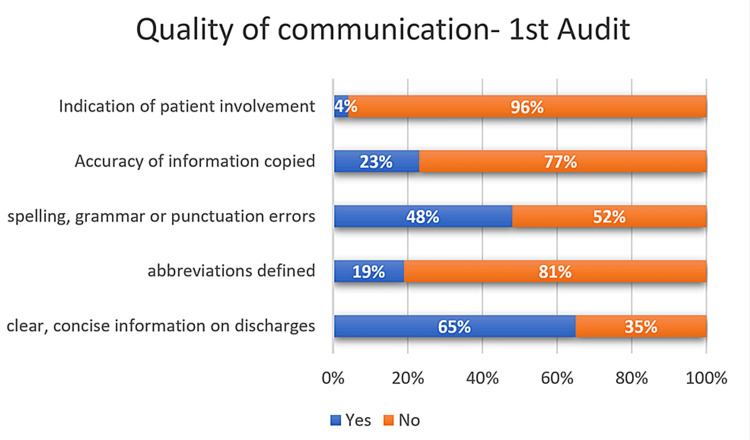
Quality of communication in discharge summaries observed in the first audit cycle

Five parameters of the DS were assessed to analyze the quality of communication in the DS. Patients were involved in the decision-making in only 4% of the DS. Abbreviations were defined in 19% of the DS. Spelling, grammar, and punctuation mistakes were present in 48% of the DS. The quality of communication observed in various aspects of DS during the first cycle is shown in Figure 5.

Quality of communication in DS observed in the RAC

**Figure 6:**
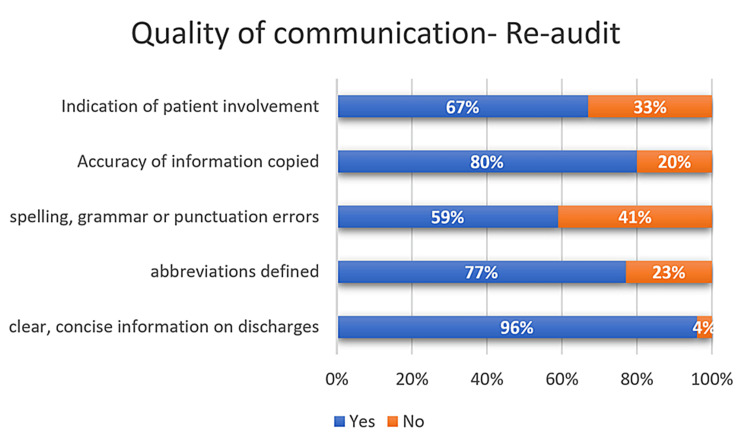
Quality of communication observed in the re-audit

All aspects of the communication were seen to have improved in the re-audit cycle of this QIP. Abbreviations were defined in 77% of the DS. Spelling, grammar, and punctuation mistakes were present in 59% of the DS. The quality of communication observed in various aspects of DS during the RAC is shown in Figure 6.

Statistical comparison between the first AC and RAC regarding the completeness of DS

During the first AC, it was observed that patients' complete name was written in 39% (n=59) of the DS; this improved to 99% (n=99) during the re-audit with a p-value of <0.05. Relevant past medical history was included in 52% (n=79) of the DS as observed during the first AC; this improved to 66% (n=66) in the RAC with a p-value of <0.05. There was no significant improvement in the documentation of primary diagnosis during the RAC (p-value=0.53). The rest of the details of the comparison between the first AC and RAC regarding the completeness of patient, admission, diagnosis, and discharge details are given in Table 1.

**Table 1:** Statistical comparison between the first audit and re-audit regarding the completeness of discharge summaries

Aspects of discharge summaries	First audit, n=150 (%)	Re-audit, n=100 (%)	Chi-squared value	p-value
The patient's complete name is written	59 (39.3%)	99 (99%)	91.84	<0.05
Patient's date of birth written	3 (2%)	66 (66%)	122.98	<0.05
Relevant past medical history included	79 (52.7%)	88 (88%)	33.77	<0.05
Primary diagnosis accurately documented	147 (98%)	99 (99%)	0.39	0.53
Secondary diagnoses written	82 (54.7%)	71 (71%)	6.74	<0.05
Hospital consultant and specialty be identified	104 (69.3%)	89 (89%)	13.18	<0.05
Details of relevant investigations have been included	90 (60%)	88 (88%)	22.94	<0.05
Post-discharge management plan clear	136 (90.7%)	98 (98%)	5.38	<0.05
Follow-up plan mentioned	98 (65.3%)	93 (93%)	22.89	<0.05

Statistical comparison between the first AC and RAC regarding the completeness of medications, allergies, and the quality of communication

The clinician completing the DS was accurately recorded in 75% (n=113) of the DS during the first AC and in 92% (n=92) of the DS during the RAC (p-value<0.05). Agents that may cause allergy and corresponding adverse reactions were recorded in 2.6% (n=4) of the DS during the first AC and in 48% (n=48) of the DS during the RAC (p-value<0.05). Abbreviations were avoided or defined in 18.6% (n=28) of the DS during the first audit and in 77% (n=77) of the DS during the RAC (p-value<0.05). The rest of the details of the comparison between the first AC and RAC regarding the completeness of medications, allergies, and quality of communication have been depicted in Table 2.

**Table 2:** Statistical comparison between the first audit and re-audit regarding the completeness of medications, allergies, and the quality of communication

Aspects of discharge summaries	First audit, n=150 (%)	Re-audit, n=100 (%)	Chi-squared value	p-value
Medication on discharge is fully documented	137 (91.3%)	99 (99%)	6.67	<0.05
Changes to the patient's medications highlighted	20 (13.3%)	87 (87%)	133.00	<0.05
Allergies recorded	4 (2.7%)	48 (48%)	77.10	<0.05
The clinician completing the discharge recorded	113 (75.3%)	92 (92%)	11.29	<0.05
All the relevant recipients have been listed	13 (8.7%)	61 (61%)	33.51	<0.05
Information is presented in a clear, concise manner	97 (64.7%)	96 (96%)	33.46	<0.05
Abbreviations have been avoided or defined	28 (18.7%)	77 (77%)	83.81	<0.05
No spelling, grammar, or punctuation errors	77 (51.3%)	59 (59%)	1.42	0.23
Information copied and pasted makes sense in isolation	35 (23.3%)	80 (80%)	77.56	<0.05
A discharge summary indicates where the patient has been involved in planning	6 (4%)	67 (67%)	115.19	<0.05

## Discussion

Accurate and comprehensive discharge information plays a vital role in ensuring patient safety and facilitating effective healthcare delivery post-discharge [9,10]. However, in practice, there are variations in the quality and quantity of discharge documentation, potentially impacting both patient safety and patient comprehension. The obstacles to an efficient discharge reported by medical professionals, nurses, patients, and their families include subpar information exchange, inadequate care coordination, and insufficient communication between hospital and care providers [11]. This quality improvement project was conducted to evaluate the impact of local, cost-effective interventions on the quality and accuracy of discharge summaries. This study highlighted the deficiencies of discharge summaries in a tertiary care hospital in Pakistan. The first audit cycle revealed inadequacies in the documentation of most components of DS. The areas most deficient included patient details, allergy details, concerns and expectations of the patients, and indications of patient involvement. Schwarz et al. discovered in their analysis of DS that optional yet important items were often lacking [12]. 

Doctors voiced concerns regarding inaccuracies and omissions in discharge summaries across multiple fields. The information gaps and mistakes that were frequently encountered include changes in medication, pending investigations, referrals, and patient or family counseling/education [2, 12-14]. Substantial improvements were observed across various components of the summary during the re-audit conducted after the implementation of interventions. The most notable difference was noted in the quality of the information in discharges, including a listing of the recipient of DS, details of allergies, written concerns and expectations of the patient, and operations/procedures written. Quality of communication was also improved in the re-audit, particularly indications of patient involvement, the accuracy of information copied, and definitions of abbreviations. Quality of communication is an often overlooked aspect of the discharge process. One study reported that the verbal exchange between patients and doctors at the time of discharge from the emergency department was often brief and incomplete, and another discovered that up to 42% of the instructions given to the patients at the time of discharge were incomplete [15]. Many patients do not understand their discharge medications and are often unable to recall their primary diagnosis [16]. This poor quality of communication during discharge leads to increased hospital readmissions and utilization [17]. The use of medical abbreviations within discharge summaries leads to confusion for both medical practitioners and patients alike. As many as 77% of physicians regarded abbreviations as undesirable elements of these summaries [18]. Therefore, it is advisable to refrain from using unclear abbreviations to enhance the comprehensibility of discharge summaries.

Major categories like patient details, relevant past medical history, secondary diagnosis, and details of patient medications were also improved significantly. Wimsett et al. concluded that discharge diagnosis, discharge medications, investigation, results, and follow-up plan were the most vital components of DS [19]. One study unveiled that 49% of discharge summaries had at least one error, and another discovered that discrepancies were present in as much as 11% of patient medication orders at discharge [20]. The literature also found a positive association between incorrect and incomplete communication of medications at discharge and adverse drug reactions [2,21]. We discovered that our educational workshop produced a significant improvement in the quality of DS being produced by the doctors at our hospital. However, this QIP had some limitations that we believe warrant a mention. Owing to the lack of similar audits in the region of our study, there was insufficient data to compare our findings with the discharge practices of Pakistan. Additionally, as this project was undertaken at the medical ward, it failed to evaluate the quality of DS at other specialties, nor were doctors from those specialties included in the workshop. Another limitation was that patient understanding of the DS and discharge instructions was not evaluated; assessment of patient satisfaction would have contributed significantly to this project. After a careful assessment of our project's results and similar studies conducted in other regions, we have set forth a list of recommendations to improve the quality of discharge summaries. We recommend the implementation of e-discharges with a unified template to reduce the chances of omission of details. We also recommend conducting additional workshops involving all specialties to enhance the overall quality of discharge summaries in the hospital.

## Conclusions

The discharge summaries (DS) were found to be incomplete and of poor quality after analyzing the results of the first cycle of this study. The details related to allergies, medications, operations, and procedures were found to be missing in most of the cases. No mention of the patient's concerns or expectations was made in the DS. The results of the re-audit showed improvement in the level of completeness of DS. The majority of the weka points observed after the first audit cycle seemed to have been improved after the re-audit, which shows that intervention proved to be quite effective in improving the completeness and quality of DS.
